# Total and Abdominal Adiposity and Hypertension in Indigenous Women in Midwest Brazil

**DOI:** 10.1371/journal.pone.0155528

**Published:** 2016-06-13

**Authors:** Juliana Barros Almeida, Kauhana Oliveira Kian, Rosangela Costa Lima, Maria Cristina Corrêa de Souza

**Affiliations:** College of Health Sciences, Federal University of Grande Dourados, Dourados, Mato Grosso do Sul, Brazil; Geisel School of Medicine at Dartmouth College, UNITED STATES

## Abstract

**Background:**

Obesity is a major risk factor for hypertension, and abdominal fat in particular has been more strongly associated with cardiovascular diseases and its prevalence has increased in Brazilian indigenous populations.

**Objective:**

The objective of this study was to estimate the prevalence of hypertension among indigenous women and its association with total and abdominal obesity after adjustment for confounding factors.

**Methods:**

This cross-sectional study evaluated indigenous non-pregnant women aged 20–59 years living in two villages of the indigenous reserve of Dourados, state of Mato Grosso do Sul, Brazil. Data were collected by trained interviewers. Households were visited and were selected by simple random sampling using SPSS software version 21. The casting of lots was performed from a list of households located on a map of villages. To locate the selected households, a Global Positioning System device was used. A questionnaire was used to obtain data on socio-demographic, lifestyle and health-related variables and to obtain anthropometric data on weight, height, and waist circumference (WC). Blood pressure was measured twice during home visits.

**Results:**

Data were collected between June and October 2013 with 362 women. Most of them were aged <40 years (66.3%) and had low educational level (≤4 years of schooling, 82.0%), had overweight/obesity (74.0%), WC ≥80 cm (83.7%), and family history of hypertension (60.5%). The prevalence of hypertension was 42.0% (CI 95%: 37.0–47.2). In the multivariable analysis, respondents with WC 80–87 cm and ≥88 cm showed approximately 2 times higher prevalence rates of hypertension compared with those with WC <80 cm after adjusting for confounding factors. There was no association between body mass index (BMI) and hypertension in this study.

**Conclusions:**

The overall prevalence of hypertension was high and associated only with abdominal adiposity but not with BMI.

## Introduction

Hypertension is the main risk factor for mortality and is responsible for 13% of all deaths worldwide [1]. Its prevalence varies widely worldwide; in Brazil, it is estimated that 24.0% of women (CI 95%: 23.7–24.4%) and 17.3% (17.0%–17.6%) of men have been diagnosed with hypertension [2]. Among the Brazilian indigenous population, it is estimated that 13.1% of women have high blood pressure [3].

One of the main risk factors for hypertension is obesity, which has increased dramatically on a global scale [4], even among the Brazilian indigenous population [3, 5, 6]. Abdominal fat in particular is strongly associated with cardiovascular and other chronic diseases and is a strong predictor of cardiovascular risk [7]. However, to the best of our knowledge, no previous studies have evaluated the association between obesity and hypertension in indigenous populations in Brazil.

Brazil has a great diversity of indigenous people (305) with large populations, represented by 817,000 indigenous individuals, corresponding to approximately 0.4% of the total population and occupying 12.5% of the Brazilian territory [8]. The State of Mato Grosso do Sul concentrates the second largest indigenous population in the country (73,295 individuals), and 15% of this population inhabits the indigenous reserve of Dourados [8]. The indigenous reserve of Dourados is composed of two villages that are primarily inhabited by ethnic group is Guarani and Terena. The area of the reserve accounts for 3539 hectares and its territorial limits are adjacent to urban areas of the municipality; thus, these indigenous groups are greatly affected by the non-indigenous population [9].

The Brazilian indigenous population is facing a rapid health transition, which involves the emergence of chronic diseases [3, 5, 6]. In this respect, a study conducted with Brazilian indigenous populations in early 1990s [10] indicated the absence of obesity and hypertension in this group, as opposed to results of recent studies conducted in different regions of the country [3, 11, 12].

The culture change process experienced by this population seems to increase risk factors for such diseases, considering that indigenous groups living in more isolated areas were less likely of developing obesity and hypertension compared with those living in less isolated areas [13].

Considering the increased prevalence of obesity and hypertension and the changes in the lifestyles of indigenous groups due to the proximity of villages to urban centres, assessing the prevalence of hypertension and associated factors is essential for the development of strategies to prevent cardiovascular diseases in these ethnic groups. Therefore, the aim of this study was to estimate the prevalence of hypertension among indigenous women living in the villages of Bororó and Jaguapirú in the municipality of Dourados, state of Mato Grosso do Sul, Brazil, and its association with total and abdominal adiposity after adjustment for confounding factors.

## Material and Methods

### Study design and sample

This population-based cross-sectional study evaluated indigenous women aged 20–59 years. Pregnant women were excluded from the study.

Hypertension prevalence rates estimated at 17.5% for indigenous people in Midwestern Brazil [3] and 29.5% for indigenous people in the village of Jaguapirú [14] were considered reference values for the sample size calculation. Assuming a prevalence of 20% with accuracy of 5% and confidence interval (CI) of 95%, the required sample size was estimated at 236 individuals. The sample size was added of 20% for losses and refusals, totalling 283 individuals.

This study is part of the project entitled "Health and nutritional profiles of indigenous people living in Dourados, state of Mato Grosso do Sul, Brazil”. Approximately 500 households were visited and were selected by simple random sampling using SPSS software version 21 (SPSS, Chicago, Illinois, USA). Only 435 (87%) households were included because the others were disabled or uninhabited. All eligible women living in households were invited to participate. Twenty-four (6.6%) women refused to participate and in 26 households, residents were not found after three visits.

The casting of lots was performed from a list of households located on a map of villages [15]. To locate the selected households, a Global Positioning System (GPS) device was used (Garmin eTrex®, Kansas, USA).

The study was approved by the Ethics Research Committee of the Federal University of Grande Dourados (Comitê de Ética em Pesquisa da Universidade Federal da Grande Dourados) under protocol No. 009/2011 and by the National Committee for Ethics in Research (Comissão Nacional de Ética em Pesquisa) under protocol No. 653/2011. The leaders of the ethnic groups and the District Council of Indigenous Health of Dourados (Conselho Distrital de Saúde Indígena de Dourados) agreed with the study and authorised the entry on indigenous territories. Women who agreed to participate signed the informed consent form.

### Data collection

Two standardised and pre-coded questionnaires prepared using data collected in the 1^st^ Brazilian Survey of Indigenous Health were used [3].

The household questionnaire inquired about the source of family income, the source of food products consumed, and number of durable goods for the assessment of the socioeconomic status (for a total of 19 items, including radio, refrigerator or freezer, Videocassete recorder [VCR] or DVD player [Digital Versatile Disc], gas cooker, washing machine, microwave oven, landline phone, mobile phone, computer, chainsaw, motorcycle/scooter, working animals, outboard engines, televisions, automobiles, air-conditioners, satellite dishes, bicycles, and cassava graters).

Socioeconomic status was evaluated using principal component analysis based on a correlation matrix of 18 durable goods (cassava grater was excluded from the analysis because it was absent in all households evaluated). This technique has been described by Coimbra Jr. et al. [3]. The resulting analysis score was classified into three categories divided according to tertiles (the 1^st^ tertile indicated the lowest socioeconomic status). The 1^st^ tertile varies from 0 to 2.53, the 2^nd^ from 2.54 to 3.99 and the 3^rd^ from 4.02 to 13.77.

The questionnaire collected data on demographic variables (residence in the villages of Bororó or Jaguapirú; age in years, 20–29, 30–39, 40–49, and >50; education in years, no education, 1–4, 5–9 and >10; socioeconomic status); lifestyle (alcohol use and smoking); and health parameters (weight, height, waist circumference [WC], family history of hypertension, blood pressure, and use of antihypertensive drugs). In addition, the source of food products consumed, including food baskets, crops, and livestock, was investigated.

Anthropometric data on weight and height were collected with individuals wearing light clothes and barefoot, according to recommendations of Lohman et al. [16]. Weight was measured using a 200 kg capacity portable digital scale with a non-slip rubber base (Marte®, model LC200PS). Height was measured using portable stadiometer with accuracy of 1 mm and field of use of 0.35–2.13 m (Alturexata®). Body mass index (BMI) was calculated by dividing weight in kilograms by the square of height in meters. The cut-off points for BMI classification were those proposed by the World Health Organization (WHO) [17].

WC was measured with a 2 m non-stretchable measuring tape with accuracy of 0.1 mm (Cescorf®) at the midpoint between the iliac crest and the lower costal margin [18]. The cut-off points for WC values considered for cardiovascular risk among women were 80.0–87.9 cm for high risk and ≥88 cm for very high risk [17].

Blood pressure was measured three times during home visits: two at the beginning and one at the end of the interview, with an interval of at least 5 minutes between the two last measurements. The first measurement was discarded. An adequately calibrated automatic digital wrist blood pressure monitor (Omron®, model HEM-631 INT) was used according to manufacturer's instructions. Blood pressure was measured with participants in the seated position, feet flat on the floor, and left arm relaxed (resting on the table or on the right arm) at heart height with the palm of the hand facing the chest. It was ensured that all participants had empty bladder, had not smoked, had not performed any physical activity, and had not consumed coffee or alcohol 30 minutes prior to evaluation. Participants were required to remain still and silent during measurements.

Hypertensive women included those who reported using antihypertensive medication and those with blood pressure levels indicative of hypertension (systolic blood pressure [SBP]≥140 mm Hg and/or diastolic blood pressure [DBP]≥90 mm Hg) [19], considering the average of two last measurements.

Interviewers were trained in workshops for the proper measurement of biometric data and completion of the questionnaire; whenever necessary, an indigenous translator, also trained, applied the questionnaires.

The standardised, pre-coded questionnaire was individually applied to all women living in the same household. In the absence of one or more household members in this age group, the interviewer would return up to three times to the same household before considering it a sample loss.

A pilot study was conducted in an indigenous village with ten women not selected in the sample to assess the questionnaire adequacy for the study objectives.

### Statistical analysis

Questionnaires were coded by interviewers and were reviewed and tabulated by the supervisor. The database was double entered in EpiData software version 3.1.

Data were analysed using STATA software version 13.0 (Stata Corporation, College Station, Texas, USA). Initially, variables were described using means and percentages and the chi-square test, Fisher's exact test, and chi-square test for linear trends. Subsequently, associations between BMI, WC, and hypertension were assessed according to the other variables. Poisson regression with robust variance was used to verify the presence of associations. In the multivariate analysis, all variables that showed associations with p<0.20 using the Wald test were included in the model. Associations with p<0.05 were considered significant.

## Results

Data were collected between June and October 2013. A total of 362 women aged 20–59 years were evaluated. Of these, 76.8% belonged to the Guarani ethnic group, and approximately 50% lived in the village of Bororó.

The mean age of the study population was 35.5 years (CI 95%: 34.4–36.6 years), and the predominant age groups were 20–29 (32.9%) and 30–39 years (33.4%). The smallest age group was 50–59 years (13.0%). The age distribution of participants was similar to that of the village population. Most respondents (82.0%) had low educational level, i.e., ≤4 years of schooling, and approximately 90.0% did not smoke or use alcohol (**Table 1**).

**Table 1:** Body mass index (BMI), waist circumference (WC), and hypertension of indigenous women living in Dourados, MS, Brazil, 2013.

Variables	Total	BMI (≥25 kg/m^2^)		WC (≥80 cm)		Hypertension	
	N (%)	N (%)	p-value	N (%)	p-value	N (%)	p-value
**Village**			0.910^a^		0.255^b^		0.025 ^b^
Bororó	174 (48.1)	128 (73.6)		149 (86.1)		84 (48.3)	
Jaguapirú	188 (51.9)	140 (74.5)		153 (81.4)		68 (36.2)	
**Ethnic group**			1.000 ^b^		0.018 ^b^		<0.001 ^b^
Guarani	278 (76.8)	206 (74.1)		239 (86.3)		132 (47.5)	
Terena	84 (23.2)	62 (73.8)		63 (75.0)		20 (23.8)	
**Age (years)**			<0.001 ^a^ 0.010^c^		<0.001 ^a^ <0.001^c^		<0.001 ^a^ <0.001^c^
20–29	119 (32.9)	72 (60.5)		80 (67.2)		21 (17.6)	
30–39	121 (33.4)	99 (81.8)		109 (90.9)		42 (34.7)	
40–49	75 (20.7)	64 (85.3)		71 (94.7)		49 (65.3)	
50–59	47 (13.0)	33 (70.2)		42 (89.4)		40 (85.1)	
**Education (years)**			0.166 ^a^ 0.980^b^		<0.001 ^a^ <0.001^b^		<0.001 ^a^ <0.001^b^
None	54 (14.9)	40 (74.1)		49 (90.7)		46 (85.2)	
1–4	243 (67.1)	186 (76.5)		207 (85.6)		97 (39.9)	
5–9	50 (13.8)	34 (68.0)		39 (78.0)		8 (16.0)	
>10	15 (4.1)	8 (53.3)		7 (46.7)		1 (6.7)	
**Socioeconomic status**			0.342^a^ 0.268^c^		0.422^a^ 0.353^c^		<0.001^a^ <0.001^c^
1^st^ tertile	127 (35.1)	81 (69.3)		99 (84.6)		66 (56.4)	
2^nd^ tertile	118 (32.6)	91 (77.1)		101 (86.3)		51 (43.2)	
3^rd^ tertile	117 (32.3)	96 (75.6)		102 (80.3)		35 (27.5)	
**Alcohol consumption**			0.852^b^		0.826^b^		0.402 ^a^
Yes	41 (11.3)	30 (73.1)		34 (82.9)		20 (48.8)	
No	321 (88.7)	238 (74.1)		269 (83.8)		132 (41.1)	
**Smoking**			0.201^b^		0.828 ^a^		0.420^b^
Yes	44 (12.2)	29 (65.9)		38 (86.4)		21 (47.7)	
No	318 (87.8)	239 (75.2)		264 (83.3)		131 (41.2)	
**BMI (kg/m**^**2**^**)**					<0.001 ^a^ <0.001^c^		0.039 ^a^ 0.044^c^
<25	94 (26.0)			39 (41.9)		29 (30.9)	
25–29.9	149 (41.2)			144 (96.6)		69 (46.3)	
>30	119 (32.9)			119 (100.0)		54 (45.4)	
**WC (cm)**			<0.001 ^a^ <0.001^c^				<0.001 ^a^ <0.001^c^
<80	59 (16.3)	5 (8.5)				9 (15.3)	
80–87	86 (23.8)	57 (66.3)				35 (40.7)	
≥88	217 (59.9)	206 (94.9)				107 (49.8)	

^a^ Chi-square test.rf

^b^Fisher’s exact test.

^c^Chi-square test for linear trends.

Data are expressed as prevalence rates.

Most respondents were overweight (74.1%), represented by 41.2% overweight and 32.9% obese individuals (Table 1). The mean BMI was 28.1 kg/m^2^ (95% CI: 27.6–28.6). The mean waist circumference (WC) was 90.9 cm (95% CI: 89.7–92.1); this variable exceeded the cut-off point of 80 cm in 83.7% of respondents.

The overall hypertension prevalence was 42.0% (95% CI: 37.0–47.2). More than half of women (60.5%) had family history of hypertension, and a small percentage of women (15.7%) reported the use of antihypertensive drugs. Of the 57 women reporting the use of antihypertensive drugs, only 15.8% presented SBP and DBP values within normal limits.

The profile of respondents stratified by BMI, WC, and hypertension are presented in Tables 1 and 2. As age increased, BMI and WC increased significantly (p<0.001) except for BMI in the age group 50–59 years. WC among women with lower educational level was 30% larger compared with those with ≥10 years of schooling.

**Table 2:** Crude prevalence ratios of body mass index (BMI), waist circumference (WC), and hypertension as functions of demographic characteristics, socioeconomic status, lifestyle, and health status of indigenous women living in Dourados, MS, Brazil, 2013.

Characteristics	BMI (≥25 kg/m^2^)		WC (≥80 cm)		Hypertension	
	PR (95% CI)	P-value^a^	PR (95% CI)	P-value ^a^	PR (95% CI)	P-value ^a^
**Village**		0.845		0.221		0.021
Bororó	1		1		1	
Jaguapirú	1.01 (0.95–1.06)		0.97 (0.94–1.02)		0.75 (0.59–0.96)	
**Ethnic group**		0.958		0.033		<0.001
Guarani	1		1		1	
Terena	1.00 (0.94–1.07)		1.06 (1.00–1.13)		1.99 (1.33–2.98)	
**Age (years)**		<0.001		<0.001		<0.001
20–29	1		1		1	
30–39	1.13 (1.06–1.21)	<0.001	1.14 (1.08–1.21)	<0.001	1.97 (1.24–3.11)	<0.001
40–49	1.15 (1.08–1.24)	<0.001	1.16 (1.10–1.23)	<0.001	3.70 (2.43–5.65)	<0.001
50–59	1.06 (0.96–1.17)	0.223	1.13 (1.06–1.21)	<0.001	4.82 (3.21–7.24)	<0.001
**Education (years)**		0.276		0.015		<0.001
None	1.14 (0.95–1.36)	0.163	1.30 (1.09–1.55)	<0.001	12.78 (1.91–85.38)	<0.001
1–4	1.15 (0.97–1.36)	0.099	1.27 (1.06–1.51)	<0.001	5.99 (0.89–40.13)	0.065
5–9	1.10 (0.91–1.31)	0.325	1.21 (1.00–1.46)	0.039	2.40 (0.32–17.73)	0.391
>10	1		1		1	
**Socioeconomic status**		0.365		0.440		<0.001
1^st^ tertile	1		1		1	
2^nd^ tertile	1.05 (0.98–1.12)	0.173	1.01 (0.96–1.06)	0.711	0.77 (0.59–1.00)	0.046
3^rd^ tertile	1.04 (0.97–1.11)	0.268	0.98 (0.93–1.03)	0.377	0.49 (0.35–0.68)	<0.001
**Alcohol consumption**		0.895		0.895		0.325
Yes	1		1		1	
No	0.99 (0.92–1.08)		1.00 (0.93–1.06)		1.19 (0.84–1.67)	
**Smoking**		0.231		0.579		0.391
Yes	1		1		1	
No	0.95 (0.87–1.04)		1.02 (0.96–1.08)		1.16 (0.83–1.62)	
**BMI (kg/m**^**2**^**)**				<0.001		0.061
<25			1		1	
25–29.9			1.39 (1.29–1.49)	<0.001	1.50 (1.06–2.13)	0.023
>30			1.41 (1.31–1.51)	<0.001	1.47 (1.02–2.11)	0.037
**WC (cm)**		<0.001				<0.001
<80	1		1		1	
80–87	1.53 (1.40–1.68)	<0.001			2.67 (1.39–5.13)	<0.001
≥88	1.80 (1.68–1.93)	<0.001			3.25 (1.75–6.02)	<0.001

95% CI = 95% confidence interval.

^a^Wald test.

PR = prevalence ratio.

Respondents with BMI ≥30 kg/m^2^ (obese) presented WC 1.41 times larger compared with those with BMI <25 kg/m^2^. Respondents with BMI between 25 and 29 kg/m^2^ (overweight) presented WC 1.39 times larger compared with those with BMI <25 kg/m^2^ (**Table 2**).

Respondents with WC ≥88 cm (i.e., very high risk for cardiovascular disease) showed 1.80 times higher prevalence of overweight and obesity compared with those with WC <80 cm, whereas those with WC of 80–87 cm (i.e., high risk for cardiovascular disease) showed 1.53 times higher prevalence of overweight and obesity compared with those with WC <80 cm. When women with WC> 80 cm were evaluated, indigenous women belonging to Terena ethnicity presented prevalence 6% higher.

With regard to hypertension, respondents from the village of Jaguapirú had 25% lower hypertension prevalence compared to those from the village of Bororó. Indigenous women belonging to the Terena ethnic group showed approximately two timers higher prevalence of hypertension compared with those belonging to the Guarani ethnic group. The increased prevalence of hypertension was proportional to the increase in age and inversely proportional to schooling. Respondents who had never attended school showed approximately 13 times higher prevalence of hypertension compared with those with ≥10 years of schooling.

As the socioeconomic status increased, the prevalence of hypertension decreased. Respondents whose socioeconomic status was in the 2^nd^ or 3^rd^ tertiles had 23% and 51% lower prevalence of hypertension, respectively, compared with those with lower socioeconomic status (1^st^ tertile).

Overweight or obese respondents showed approximately 50% higher hypertension prevalence compared with those with low or normal weight. Respondents with WC 80–87 cm and ≥88 cm were 2.67 and 3.25 times more likely of having higher prevalence of hypertension, respectively, compared with those with WC <80 cm. The other variables showed no statistically significant differences (Table 2).

**Table 3**shows the results of the multivariate analysis. The prevalence of hypertension was proportional to the increase in WC (p = 0.032). Respondents with WC 80–87 cm and ≥88 cm were approximately 2 times more likely of having higher prevalence of hypertension compared with those with WC <80 cm after adjusting for the place of residence (village), age, BMI, and education. BMI showed no statistically significant differences with hypertension in this study.

**Table 3:** Prevalence ratios of hypertension as a function of body mass index (BMI) and waist circumference (WC) after adjustment for confounding factors among indigenous women living in Dourados, MS, Brazil, 2013.

Characteristics	Hypertension	
	PR (95% CI)	p-value^c^
**BMI (kg/m**^**2**^**)**^**a**^		0.788
<25	1	
25–29.9	0.93 (0.66–1.32)	0.693
>30	0.88 (0.59–1.30)	0.509
**WC (cm)**^**b**^		0.032
<80	1	
80–87	1.84 (1.05–3.23)	0.033
≥88	2.18 (1.21–3.91)	<0.001

95% CI = 95% confidence interval.

^a^BMI: adjusted for age and WC.

^b^WC: adjusted for location (village), age, education, and BMI.

^c^Wald test; PR = prevalence ratio.

## Discussion

This study found high prevalence of hypertension (42.0%), overweight/obesity (74.1%) and WC ≥80 cm (83.7%).

The prevalence of hypertension among indigenous women from both indigenous villages of Dourados, MS (42.0%) evaluated was higher than the prevalence rates estimated for indigenous women (13.2%) [3] and non-indigenous women (24.0%) [2] both on a national scale and for the Midwestern region of Brazil (17.5%) [3]. In addition, these values were higher than those obtained for indigenous women in Mexico (6.8%) [20] but lower than those obtained for indigenous women in villages in India (50.5%) [21].

The high prevalence of hypertension reflects the transition process experienced by these ethnic groups, combined with the difficulty inherent in surviving in accordance with their traditions given their territorial confinement. The guarantee of land tenure for indigenous people ensures not only their subsistence but also their social and cultural continuity [22].

A survey conducted in the 1980s with Brazilian Indians who lived in isolated regions indicated the absence of hypertension [10]. This finding may be associated with their diet, which is based on the consumption of food products obtained from hunting, fishing, and agriculture, and the limited consumption of salt and processed foods.

One aspect that can contribute to the westernisation of the eating habits of indigenous populations of Dourados is the proximity of the reserve to the urban centre and the consumption of low-cost food products, with high contents of carbohydrates, lipids and calorie. Accordingly, indigenous populations living in more isolated areas are less likely of presenting diseases such as hypertension and obesity compared with those living in less isolated areas [13].

Ethnic differences in the prevalence of hypertension were found, being higher in Terena ethnicity. This may be due to the characteristics of body weight accumulation, changes in lifestyle that include physical activity and nutrition, genetic factors inherent to ethnicity and cultural changes experienced by these populations [23].

The prevalence of hypertension increases with advancing age in modernized populations. This association is primarily responsible for the increased incidence and prevalence of hypertension [19]. In the present study, a higher prevalence of hypertension was observed among older women; this result was similar to that observed for non-indigenous women in Brazil [24].

However, a study on Yanomámi Indians and with other isolated indigenous people from the state of Rondônia indicated no association between increased blood pressure and increasing age [10, 25]. Suruí Indians presented weak association between SBP and age [11]. The distinct results observed may be related to the different levels of assimilation of western culture experienced by these indigenous groups. It is noteworthy that cohort effects, i.e., changes in population exposure to risk factors over time can affect the prevalence of the disease.

The results of this study indicate that indigenous women with lower socioeconomic status and those with lower educational level have higher prevalence of hypertension. A previous study has reported that the prevalence rates of chronic diseases tended to be higher among individuals of lower socioeconomic status [26] and Suruí Indians showed higher prevalence of hypertension [11]. In this respect, individuals with lower educational level and lower socioeconomic status generally have poorer health status [27].

Although blood pressure levels are correlated among family members due to common genetic background [28] like those observed in study in the same region [12], this study found no significant association between family history and hypertension.

Among women with previous diagnosis of hypertension who reported the use of antihypertensive drugs, 84% had altered blood pressure levels. Women in this condition may represent the proportion of the population receiving inadequate antihypertensive treatment. Considering the fact that uncontrolled hypertension increases the vulnerability for the development of cardiovascular events, the findings of present study are important. Low and middle-income countries have high prevalence of individuals with uncontrolled hypertension compared to high-income countries [29].

BMI analysis indicated excess weight among 74.1% of indigenous women from villages of Dourados, MS. In this study, excess weight was higher than the average values observed for both non-indigenous women (47.4%) [24] and indigenous women in Brazil (45.9%) [3].

Previous studies on distinct indigenous communities in Brazil have reported high rates of overweight and obesity rates. However, the prevalence ratios reported in this study were higher than those previously reported. A study on BMI among Xingu Indians reported prevalence of excess weight of 58.5% in the age group 20–49 years and 47.6% in the age group older than 50 years among women [5]. Another study on the Suruí Indians reported that 42.3% of adults aged <50 years were overweight and 18.2% were obese. However, the obesity rate among women was twice that among men [6].

Other studies on the nutritional status of the Brazilian population have shown a continuous increase in the prevalence of overweight. A national survey conducted in 2013 reported increases of 8.9% in overweight and 5.4% in obesity among women in 7 years [24].

This study found that the prevalence of overweight tended to increase with age until the age of 49 years; like a result similar to that observed in another study conducted in Brazil [24].

With regard to WC, the prevalence of excess abdominal adiposity (WC ≥80 cm) was 83.7%, higher in ethnicity Terena. Jannsen et al. [30] reported that the health risks related to excess body fat are mainly due to abdominal adiposity. Abdominal obesity was detected in 61.0% of female Suya Indians [31]. Similarly, among Xingu Indians, the prevalence of abdominal obesity was higher among women compared to men (76.4% vs. 22.6%) [6]. However, Stoddard et al. [32], found dissimilar results and suggest the presence of a protective factor against obesity and metabolic disorders in Mexican indigenous. These denote possible differences among ethnic groups and the need for further studies.

The excess abdominal adiposity observed in indigenous women was higher among those with lower educational level. Similarly, the rates observed among non-indigenous Brazilian women indicate a similar association between overweight and educational level [24].

Recent studies have emphasised the transition process that indigenous groups are experiencing, which contributes to a situation of dietary and nutritional vulnerability due to environmental and socioeconomic changes to which these groups are exposed [5,33].

The changes in the health and nutritional status of indigenous groups seem to have initiated with their closer contact with western society, suggesting that this proximity has a negative effect on their lifestyle and favours the emergence of chronic diseases [6–25], like the results of this study that showed high prevalence of hypertension and adiposity.

The hypertension rate attributable to obesity is very high and has been estimated at 60.0% for women [34]. The present study found higher prevalence ratio for hypertension (1.84 and 2.18) for women with WC ≥ 80 cm and WC ≥ 88 cm, respectively, compared with women with normal WC.

The results presented here are in accordance with the information that fat accumulation in the abdominal region (visceral fat) results in increased risk of developing hypertension [35] and that WC, and not BMI, explains the obesity-related health risks [30].

Abdominal adiposity is also a strong predictor of certain comorbidities, including dyslipidaemia, metabolic syndrome, and cardiovascular diseases [7, 30, 35]. That is, high WC even in normal weight individuals leads to health risks [30, 36].

It is noteworthy that the cut-off points used in this study were not specific to indigenous populations and that, due to the cross-sectional nature of this study, it is not possible to establish a causal link. Moreover, it should be observed that the results of this study could not be extrapolated to all indigenous groups in Brazil due to the peculiarities of these groups.

Another negative point is that lifestyle risk factors such as physical activity, stress, diet and sodium, dietary fibre and fat intake were not assessed. In addition, only one visit was made to measure blood pressure. To minimize this problem, three measurements were performed and the average of the last two measures was used. Our results indicate high prevalence rates of overweight, obesity, and hypertension and an association between abdominal adiposity and hypertension among indigenous women from indigenous villages of Dourados, MS, but not BMI. However, a significant association between hypertension and total obesity was not detected when adjusting for confounding factors. Nonetheless, these results underscore the increased risks of developing metabolic syndrome, diabetes, dyslipidaemia, and other cardiovascular events in this population.

Therefore, increased efforts to implement appropriate strategies for the prevention and control of chronic diseases is essential, particularly hypertension and obesity/abdominal obesity, combined with the promotion of education to create awareness about hypertension and its control by the indigenous population of Dourados.

## References

[1] World Health Organization. Noncommunicable diseases country profiles 2011. 2011. Available: http://www.who.int/nmh/publications/ncd_profiles2011/en/.

[2] Schmidt MI, Duncan BB, Silva EA, Menezes AM, Monteiro CA, Barreto SM, et al Chronic non-communicable diseases in Brazil: burden and current challenges. Lancet. 2001;377: 1949–1961. 10.1016/S0140-6736(11)60135-9 21561658

[3] Coimbra CEA, Santos RV, Welch JR, Cardoso AM, de Souza MC, Garnelo L, et al The First National Survey of Indigenous People’s Health and Nutrition in Brazil: rationale, methodology, and overview of results. BMC Pub Health. 2013;13: 1–52. 23331985 10.1186/1471-2458-13-52 PMC3626720

[4] World Health Organization. Global health risks: mortality and burden of disease attributable to select major risks. 2009. Available: http://www.who.int/healthinfo/global_burden_disease/GlobalHealthRisks_report_full.pdf.

[5] Lourenço AEP, Santos Rv, Orellana JD, Coimbra CEA Jr. Nutrition transition in Amazonia: obesity and socioeconomic change in the Suruí Indians from Brazil. Am J Human Biol. 2008;20: 564–571. 18442078 10.1002/ajhb.20781

[6] Gimeno SG, Rodrigues D, Canó EN, Lima EE, Schaper M, Pagliaro H, et al Cardiovascular risk factors among Brazilian Karib indigenous peoples: Upper Xingu, Central Brazil, 2000–3. J Epidem Commun Health. 2009;63: 299–304. 10.1136/jech.2008.077966 19028731

[7] Zhu S, Wang Z, Heshka S, Heo M, Faith MS, Heymsfield SB. Waist circumference and obesity-associated risk factors among whites in the Third National Health and Nutrition Examination Survey: clinical action thresholds. Am J Clin Nutr. 2002;76: 743–749. 12324286 10.1093/ajcn/76.4.743

[8] Instituto Brasileiro de Geografia e Estatística: Censo Demográfico 2010: Características Gerais dos Indígenas. Resultados do Universo. 2010. Available: http://biblioteca.ibge.gov.br/visualizacao/periodicos/95/cd_2010_indigenas_universo.pdf.

[9] Santana JR Junior. Produção e reprodução indígena: o vir e o porvir na reserva de Dourados/MS. Campo-território: revista de geografia agrária. 2010;5: 203–236.

[10] Mancilha-Carvalho JJ, Silva NAS. The Yanomami indians in the INTERSALT study. Arq Bras Cardiol. 2003;80: 295–300. 10.1590/s0066-782x2003000300005 12856272

[11] Tavares FG, Junior CEAC, Cardoso AM. Níveis tensionais de adultos indígenas Suruí, Rondônia, Brasil. Rev Ciênc Saúde Col. 2013;18: 1399–1409. 23670468

[12] Oliveira GF, Oliveira TRR, Ikejiri AT, Andraus MP, Galvao TF, Silva MT, et al Prevalence of hypertension and associated factors in an indigenous community of Central Brazil: a population-based study. PloS One. 2014;9: e86278 10.1371/journal.pone.0086278 24489710 PMC3904906

[13] Roriz-Cruz M, Rosset I, Barreto-Roriz R, Mancilha-Carvalho JJ. Acculturation, obesity, and hypertension among female Brazilian Indians [letter]. Hypertension. 2010;56: e43–e44. 10.1161/HYPERTENSIONAHA.110.158956 20733085

[14] Oliveira GF, Oliveira TRR, Rodrigues FF, Corrêa LF, Ikejiri AT, Casulari LA. Prevalência de diabetes melito e tolerância à glicose diminuída nos indígenas da Aldeia Jaguapiru, Brasil. Rev Panam Salud Publica. 2011;29: 315–321. 21709935 10.1590/s1020-49892011000500003

[15] Instituto Brasileiro de Geografia e Estatística. Projeção Geográfica–Datum SAD69. Malha Digital Municipal. 2010. Available: http://mapas.ibge.gov.br/bases-e-referenciais/bases-cartograficas/malhas-digitais.

[16] Lohman TG, Roche AF, Martorell R. Anthropometric standardization reference manual. Champaign: Human Kinetics Books; 1988.

[17] World Health Organization. Obesity: Preventing and Managing the Global Epidemic. Technical Report Series, n. 894. Geneva: World Health Organization; 2000. 11234459

[18] World Health Organization. Expert Committee on physical status: the use and interpretation of anthropometry. Technical Report Series, n. 854. Geneva: World Health Organization; 1995. 8594834

[19] Joint National Committee on Prevention, Detection, Evaluation, and Treatment of High Blood Pressure. The Seventh report of the Joint National Committee on Prevention, Detection, Evaluation, and Treatment of High Blood Pressure (JNC 7). JAMA. 2003;289: 2560–2572. 12748199 10.1001/jama.289.19.2560

[20] Guerrero-Romero F, Rodriguez-Moran M, Sandoval-Herrrera F, Alvarado-Ruiz R. Prevalence of hypertension in indigenous inhabitants of traditional communities from the north of Mexico. J Human Hypert. 2000;14: 555–559. 10.1038/sj.jhh.1001067 10980586

[21] Manimunda SP, Sugunan AP, Benegal V, Balakrishna N, Rao MV, Pesala KS. Association of hypertension with risk factors & hypertension related behaviour among the aboriginal Nicobarese tribe living in Car Nicobar Island, India. Indian J Med Res. 2011;133: 287–293. 21441682 PMC3103153

[22] Santos RV, Coimbra CEA Jr. Cenários e tendências da saúde e da epidemiologia dos povos indígenas no Brasil In: Coimbra CEA Jr., Santos RV, Escobar AL, editors. Epidemiologia e saúde dos povos indígenas no Brasil. Rio de Janeiro: Fiocruz/ABRASCO; 2003 pp 1–120.

[23] Rhoades DA, Welty TK, Wang W, Yeh F, Devereux RB, Fabsitz RR, et al Aging and the Prevalence of Cardiovascular Disease Risk Factors in Older American Indians: The Strong Heart Study. J Am Geriatr Soc. 2007;55(1): 87–94. 17233690 10.1111/j.1532-5415.2006.01018.x

[24] Brasil. Ministério da Saúde. Secretaria de Vigilância em Saúde. Vigitel Brasil 2013: Vigilância de Fatores de Risco e Proteção para Doenças Crônicas por Inquérito Telefônico. Brasília: Ministério da Saúde; 2014.

[25] Pavan L, Casiglia E, Braga LMC, Winnicki M, Puato M, Pauletto P, et al Effects of a traditional lifestyle on the cardiovascular risk profile: the Amondava population of the Brazilian Amazon. Comparison with matched African, Italian and Polish populations. J Hypert. 1999;17: 749–756. 10.1097/00004872-199917060-00005 10459871

[26] Barros MBA, Francisco PMSB, Zanchetta LM, César CLG. Trends in social and demographic inequalities in the prevalence of chronic diseases in Brazil. PNAD: 2003–2008. Ciênc Saúde Coletiva. 2011;16: 3755–3768. 10.1590/s1413-81232011001000012 21987319

[27] Braveman PA, Cubbin C, Egerter S, Williams DR, Pamuk E. Socieconomic disparities in health in the United States: what the patterns tell us. Am J Public Health. 2010;100: S186–S196. 10.2105/AJPH.2009.166082 20147693 PMC2837459

[28] Joint National Committee on Prevention, Detection, Evaluation, and Treatment of High Blood Pressure. The Sixth Report of the Joint National Committee on Prevention, Detection, Evaluation, and Treatment of High Blood Pressure (JNC 6). Arch Intern Med. 1997;157: 2413–2446. 9385294 10.1001/archinte.157.21.2413

[29] World Health Organization. A global brief on Hypertension. Silent killer, global public health crisis. World Health Day 2013. 2013. Available: http://apps.who.int/iris/bitstream/10665/79059/1/WHO_DCO_WHD_2013.2_eng.pdf?ua=1.

[30] Janssen I, Katzmarzyk PT, Ross R. Waist circumference and not body mass index explains obesity-related health risk. Am J Clin Nutr. 2004;79: 379–384. 14985210 10.1093/ajcn/79.3.379

[31] Salvo VLMA, Rodrigues D, Baruzzi RG, Pagliaro H, Gimeno SGA. Perfil metabólico e antropométrico dos Suyá: Parque Indígena do Xingu, Brasil Central. Rev Bras Epidemiol. 2009;12: 458–468.

[32] Stoddard P, Handley MA, Vargas Bustamante A, Schillinger D. The influence of indigenous status and community indigenous composition on obesity and diabetes among Mexican adults. Soc Sci Med. 2011;73(11): 1635–1643. 10.1016/j.socscimed.2011.09.006 22033376

[33] Fávaro T, Ribas DLB, Zorzatto JR, Segall-Corrêa AM, Panigassi G. Segurança alimentar em famílias indígenas Teréna, Mato Grosso do Sul, Brasil Food security in Teréna indigenous families, Mato Grosso do Sul, Brazil. Cad Saúde Pública. 2007;23: 785–793. 17435876 10.1590/s0102-311x2007000400006

[34] Garrison RJ, Kannel WB, Stokes J, Castelli WP. Incidence and precursors of hypertension in young adults: the Framingham Offspring Study. Prevent Medic. 1987;16: 235–251. 10.1016/0091-7435(87)90087-9 3588564

[35] Wofford MR, Hall JE. Pathophysiology and treatment of obesity hypertension. Curr Pharmac Design. 2004;10: 3621–3637. 10.2174/1381612043382855 15579059

[36] Folsom AR, Kaye SA, Sellers TA, Hong C-P, Cerhan JR, Potter JD, et al Body fat distribution and 5-year risk of death in older women. JAMA. 1993;269: 483–487. 8419667

